# The Status of Occupational Protection During COVID-19 Pandemic: Knowledge, Attitudes, and Practice of Healthcare Workers in Endoscopy Units, China

**DOI:** 10.3389/fpubh.2021.632608

**Published:** 2021-03-22

**Authors:** Yuan Tian, Bixiao Nian, Yongchen Ma, Xinyue Guo, Feng Wang, Long Rong

**Affiliations:** Department of Endoscopy Center, Peking University First Hospital, Beijing, China

**Keywords:** occupational protection, COVID-19, endoscopy, knowledge, attitudes, practice

## Abstract

**Background:** SARS-CoV-2 spreads rapidly around the world, and some patients present gastrointestinal symptoms. The existence of the virus in the gastrointestinal tract makes digestive endoscopy a high-risk operation, which associated with an increased risk of infection rate in healthcare workers. This study aimed at exploring current knowledge, practice and attitudes of healthcare workers in endoscopy units in China regarding the status of occupational protection during COVID-19 pandemic.

**Methods:** A cross-sectional study of a national online survey involving 717 healthcare workers in endoscopy units from 94 medical structures in 24 provinces and municipalities around China was conducted online via a questionnaire platform called Wenjuanxing (wjx.cn). The data were analyzed using correlation approaches, Kruskal-Wallis test for independent samples, and linear regression models.

**Results:** Most Chinese healthcare workers in endoscopy units had a good knowledge of COVID-19 (median: 10; range: 7–12), showed a strikingly positive attitude (median: 65; range: 39–65), and carried out good practice (median: 47; range: 14–50) in strengthening the protection, disinfection and management of COVID-19. In terms of attitudes, female staff was more concerned about protection against COVID-19 than male staff (KW = 8.146, *P* = 0.004). Nurses performed better in both attitude (KW = 2.600, *P* = 0.009) and practice (KW = 6.358, *P* < 0.001) than endoscopic physicians when carrying out personal protection, patient care and environmental disinfection against SARS-CoV-2 infection. More positive attitudes in protection were related to better protective behavior in endoscopic daily medical work (*r* = 0.312; *P* < 0.001).

**Conclusion:** The findings of this study suggest that Chinese endoscopy healthcare workers have an excellent mastery of knowledge about COVID-19, which is transformed into positive beliefs and attitudes, contributing to good practice during daily endoscopic procedures. Medical staff may benefit from further education. With the gradual normalization amid the ongoing COVID-19 pandemic, protection and management in endoscopy units may be changed accordingly.

## Introduction

The severe acute respiratory syndrome caused by new coronavirus (SAS-CoV-2) was first cluster in December 2019 and reported from China (1). This disease was spread into global pandemic rapidly, and a total of 93,194,922 confirmed cases and more than 2 million deaths were reported in January 2021 (2). The pandemic of coronavirus disease 2019 (COVID-19) in China at stable status, while a “second wave” of contagion was outbreak outside of China (3). As a highly contagious disease, the risk of infection among healthcare workers is significant. Twenty nine percentage of patients (40 out of 138) were healthcare workers in one of the earliest studies in Wuhan (4). A report of American Center for Disease Control and Prevention (CDC) of US stated that from February 12 to April 9, a total of 9,282 healthcare workers were diagnosed with COVID-19, including 27 deaths. Eleven to nineteen percentage of COVID-19 cases were identified as medical staffs (5). Studies have already illustrated the virus transmission, and found physical distancing of 1 m or more, and use of face masks, respirators, and eye protection could prevent the transmission of COVID-19 (6–13) while the current knowledge, practice and attitudes of healthcare workers in endoscopy units remains unclear.

Digestive symptoms are increasingly recognized among patients with COVID-19, including anorexia, diarrhea, nausea, vomit, and abdominal pain (14). Several studies pointed out that some patients presented only GI symptoms and no typical symptoms throughout the course of the disease (15). Viral RNA was detected in the feces of COVID-19 patients, and active virus particles were isolated (16). Most atypical patients with GI symptoms did not visit the Pulmonary Department, Emergency Department or Fever Clinic, but the Gastroenterology Department, which resulted in healthcare providers being exposed to either respiratory and gastrointestinal droplets or body fluids from patients when performing endoscopy. Aerosols generated from coughing in upper endoscopy and flatus produced in colonoscopy played an important role in endoscopist exposure to the virus (17). Endoscopy therefore was a potential route of infection according to the characteristics and transmission of the virus. These preliminary findings highlight that adequate protection of healthcare workers is critical.

The theory of knowledge, attitude/belief and practice (KAP) model on PHEIC may distinguish from general issues (18, 19). At the early stage of SARS-CoV-2 epidemic in China, National Health Commission of the PRC and Chinese CDC conducted public education and took prevention measures quickly in the whole society as responses to COVID-19 (20). In addition, the Chinese Society of Digestive Endoscopy also made special regulations on endoscopic work (21). With the joint efforts, people's knowledge reserve for epidemic prevention and control reached a high and stable level, which partially accounted for the negative results from knowledge. It is easier for endoscopic healthcare workers who have received medical education for years to master the knowledge of COVID-19. For instance, endoscopy physicians who believe low population density can reduce the transmission of SARS-CoV-2 may limit the daily number of patients examined. Given the adequate protective knowledge, different attitudes lead to different practice. This cross-sectional study was performed using an online questionnaire to evaluate the occupational protection status of healthcare workers in endoscopy units of different hospital scale in different regions in China. The level of knowledge and awareness of healthcare workers about COVID-19 occupational protection during the pandemic, or the behavior of participants with respect to personal protective equipment and disinfection management were assessed in this study, so as to give advice and suggestions to endoscopic units in other regions.

## Materials and Methods

### Study Subjects

Endoscopic healthcare workers, including endoscopy physicians, nurses, and cleaning workers from general hospitals, specialized hospitals and community medical institutions from 94 medical structures in 24 provinces and municipalities around China were enrolled and invited to complete the questionnaire in this study. Ten times the number of questionnaire entries with extra 10% invalid questionnaires, 389 was regarded as the minimum sample size for this study. This study was approved by the Peking University First Hospital Biomedical Research Ethics Committee (No. 2020-124). All subjects finally enrolled in this study were considered to have signed informed consent agreement prior to answering the questionnaire.

### Questionnaire Design

Based on the guidance issued by Chinese Medical Association on the endoscopic diagnosis and treatment during the prevention and control of new coronavirus infection, the questionnaire items were designed and screened by a group of specialists who had experience in the fields of endoscopic diagnosis and treatment, epidemic prevention and control, and public health research. This questionnaire was applied to the evaluation of endoscopic healthcare workers from three aspects, namely, knowledge, attitudes, and behavior toward COVID-19. More details are shown in Table 1 and Appendix 1 (Supplementary Material). The response for each item of knowledge part was scored 0–1. A five-grade scoring method was used to indicate the level for attitude part: 5, strongly agree; 4, agree; 3, neutral; 2, disagree; 1, strongly disagree. Moreover, the five-grade scoring method was applied to indicate the level for practice part: 5. Always; 4. Often; 3. Sometimes; 2. Occasionally; 1. Hardly ever. The scoring system for knowledge ranged from 0 to 12, and the good knowledge score was defined as >7.2 (above 60%), and poor knowledge was defined as below 60%. Similarly, the scoring system for attitude and practice ranged from 13 to 65, and 10 to 50, respectively, and the good attitude and good practice were defined as > 52 (attitude scores above 80% were defined as good attitude) and > 40 (scores >80% were classified as having good practice), respectively (22).

**Table 1:** Demographic characteristics of subjects.

**Items**	**No. (n)**	**Ratio (%)**
**Gender**		
Male	206	28.7%
Female	511	71.3%
**Age**		
20–35	219	30.54%
36–50	439	61.23%
51–65	59	8.23%
**Occupational identity**		
Endoscopic physicians	329	45.9%
Nurses	378	52.7%
Cleaning workers	10	1.4%
**Length of service**		
<5 years	200	27.9%
5–10 years	277	38.6%
>10 years	240	33.5%
**Education**		
Bachelor degree or below	614	85.6%
Master degree or above	103	14.4%
**Hospital grade**		
Primary	14	2.0%
Secondary	237	33.0%
Tertiary	466	65.0%

### Questionnaire Evaluation

The quality of the present questionnaire was evaluated from two aspects, namely, validity and reliability. For content validity, the consistency of the contents to be tested with questionnaire items was assessed by five experts from related fields using a four-level scoring method, in which score 1 represented “irrelevant,” 2 “a little bit relevant,” 3 “relevant,” and 4 “very relevant.” Content validity index (CVI) was served as the measurement, and an index value of >0.8 indicated an acceptable content validity. External reliability, also known as test-retest reliability, was also examined in this study.

### Investigation Method

Electronic questionnaire was adopted in this study to investigate current situations of endoscopic healthcare workers during COVID-19 pandemic. The questionnaire entries were imported to the online platform Wenjuanxing (wjx.cn), and distributed to endoscopic healthcare workers around China via WeChat. All the subjects were invited to finish the survey before April 4th, 2020. The data were subsequently downloaded and sorted by specialists. Investigators were blinded to the identity information of the subjects.

### Statistical Analysis

Descriptive statistics was used to summarize demographic data, and internal reliability was measured by Cronbach's α. The questionnaire scores according to demographic data were compared by using independent sample *t*-test, Mann-Whitney U test, one-way analysis of variance, rank-sum test and Pearson/Spearman correlation analysis separately based on the data distribution. A *P* < 0.05 was considered to be significant, and the results of all tests noted above were analyzed using SPSS 24.0 software.

## Results

### Demographic Characteristics of Subjects

A total of 717 valid questionnaires were collected before April 4th. The questionnaire was completed by healthcare workers from 94 medical structures in 24 provinces and municipalities. More demographic details are shown in Table 1. The average rating index of this questionnaire was defined as CVI, which was 0.924, indicating an acceptable content validity.

### Level of Knowledge

The distribution of responses to the statements that examined the level of knowledge with respect to COVID-19 is presented below (Table 2). The variable ranged from 0 to 12. Overall, medical staff in endoscopy units had a good knowledge, with the median total score of 10 (total score range: 7–12), and 83.33% of accuracy. The good knowledge rate was 99.4% (713/717). There were no significant differences between other demographic characteristics and the level of knowledge about COVID-19.

**Table 2:** Distribution of responses to the knowledge questionnaire.

	**Score distribution** ***n*** **(%)**				***P*-value**
	**Median (range)**	**7–8**	**9–10**	**11–12**	
**Sex**					
Male	10 (7–12)	13 (6.31)	109 (52.91)	84 (40.78)	0.991
Female	10 (7–12)	17 (3.33)	295 (57.73)	199 (38.94)	
**Age**					
<40	10 (7–12)	17 (4.97)	190 (55.56)	135 (39.47)	0.810
≥40	10 (7–12)	13 (3.47)	214 (57.07)	148 (39.47)	
**Occupational identity**					
Endoscopic physicians	10 (7–12)	21 (6.38)	183 (55.62)	125 (37.99)	0.345
Nurses	10 (7–12)	9 (2.38)	214 (56.61)	155 (41.01)	
Cleaning workers	10 (10–11)	0 (0)	7 (70.00)	3 (30.00)	
**Length of service**					
<5 years	10 (7–12)	11 (5.50)	115 (57.50)	74 (37.00)	0.551
5–10 years	10 (7–12)	10 (3.61)	157 (56.68)	110 (39.71)	
>10 years	10 (7–12)	9 (3.75)	132 (55.00)	99 (41.25)	
**Education**					
Bachelor degree or below	10 (7–12)	23 (3.75)	340 (55.37)	251 (40.88)	0.036
Master degree or above	10 (8–12)	7 (6.80)	64 (62.14)	32 (31.07)	
**Hospital Grade**					
Primary	10 (8–11)	1 (7.14)	8 (57.14)	5 (35.71)	0.729
Secondary	10 (7–12)	11 (4.64)	127 (53.59)	99 (41.77)	
Tertiary	10 (7–12)	18 (3.86)	269 (57.72)	179 (38.41)	

### Level of Attitudes

The distribution of responses to statements that examined attitudes is shown in Table 3. The variable in attitudes ranged from 13 to 65, and medical staff had a strikingly positive attitude toward strengthening the protection, disinfection and management of COVID-19, with the median score of 65 (score range: 39–65). 99.3% (712/717) of participants supported limited daily endoscopy services or service suspension, and 92.9% (666/717) had a positive attitude toward risk-based screening before the endoscopy procedure and appropriate occupational protection during the outbreak. The good attitude rate was 99.3% (712/717). Female staff were more concerned about COVID-19 than male staff (KW = 8.146, *P* = 0.004), and the same phenomenon was observed between nurses and physicians. Nurses had a more positive attitude than physicians (KW = 2.600, *P* = 0.009, Adj. *P* = 0.028).

**Table 3:** Distribution of responses to the attitude questionnaire.

	**Score distribution** ***n*** **(%)**							***P*-value**
	**Median (range)**	**<50**	**51–53**	**54–56**	**57–59**	**60–62**	**63–65**	
**Sex**								
Male	65 (39–65)	4 (1.94)	7 (3.40)	5 (2.43)	18 (8.74)	20 (9.71)	152 (73.79)	0.004
Female	65 (51–65)	0 (0)	9 (1.76)	6 (1.17)	18 (3.52)	59 (11.55)	419 (82.00)	
**Age**								
<40	65 (39–65)	2 (0.58)	9 (2.63)	4 (1.17)	17 (4.97)	33 (9.65)	277 (80.99)	0.447
≥40	65 (44–65)	2 (0.53)	7 (1.87)	7 (1.87)	19 (5.07)	46 (12.27)	294 (78.40)	
**Occupational identity**								
Endoscopic physicians	65 (39–65)	4 (1.22)	7 (2.13)	8 (2.43)	26 (7.90)	35 (10.64)	249 (75.68)	0.023
Nurses	65 (51–65)	0 (0)	9 (2.38)	3 (0.79)	10 (2.65)	43 (11.38)	313 (82.80)	
Cleaning workers	65 (62–65)	0 (0)	0 (0)	0 (0)	0 (0)	1 (10.00)	9 (90.00)	
**Length of service**								
<5 years	65 (39–65)	2 (1.00)	6 (3.00)	2 (1.00)	8 (4.00)	21 (10.50)	161 (80.50)	0.708
5–10 years	65 (52–65)	0 (0)	8 (2.89)	4 (1.44)	16 (5.78)	25 (9.03)	224 (80.87)	
>10 years	65 (44–65)	2 (0.83)	2 (0.83)	5 (2.08)	12 (5.00)	33 (13.75)	186 (77.50)	
**Education**								
Bachelor degree or below	65 (39–65)	2 (0.33)	15 (2.44)	10 (1.63)	26 (4.23)	65 (10.59)	496 (80.78)	0.063
Master degree or above	65 (44–65)	2 (1.94)	1 (0.97)	1 (0.97)	10 (9.71)	14 (13.59)	75 (72.82)	
**Hospital Grade**								
Primary	65 (59–65)	0 (0)	0 (0)	0 (0)	1 (7.14)	1 (7.14)	12 (85.71)	0.102
Secondary	65 (39–65)	2 (0.84)	2 (0.84)	6 (2.53)	19 (8.02)	30 (12.66)	178 (75.11)	
Tertiary	65 (44–65)	2 (0.43)	14 (3.00)	5 (1.07)	16 (3.43)	48 (10.30)	381 (81.76)	

### Level of Practice

Table 4 shows the distribution of responses to statements that examined personal protection, patient care and disinfection management practice or behavior. The variable in behavior ranged from 10 to 50. The median score of the survey was 47 (score range: 14–50), which showed that medical staff had good practice in COVID-19. The good practice rate was 87.2% (625/717). The comparison of attitudes showed that 93.8% (673/717) of the subjects provided limited daily endoscopy services, the risk-based visit process was implemented in the endoscopy units of 88.1% (632/717) of the subjects, and 1.4% (10/717) believed that their hospitals needed to increase the supply of personal protective equipment.

**Table 4:** Distribution of responses to the practice questionnaire.

	**Score distribution** ***n*** **(%)**								***P*-value**
	**Median (range)**	**<20**	**21–25**	**26–30**	**31–35**	**36–40**	**41–45**	**46–50**	
**Sex**									
Male	46 (18–50)	1 (0.49)	0 (0)	6 (2.91)	12 (5.83)	32 (15.53)	47 (22.82)	108 (52.43)	0.000
Female	48 (14–50)	1 (0.20)	4 (0.78)	7 (1.37)	16 (3.13)	33 (6.46)	102 (19.96)	348 (68.10)	
**Age**									
<40	48 (18–50)	1 (0.29)	3 (0.88)	9 (2.63)	13 (3.80)	37 (10.82)	62 (18.13)	217 (63.45)	0.545
≥40	47 (14–50)	1 (0.27)	1 (0.27)	4 (1.07)	15 (4.00)	28 (7.47)	87 (23.20)	239 (63.73)	
**Occupational identity**									
Endoscopic physicians	46 (18–50)	1 (0.30)	0 (0)	9 (2.74)	18 (5.47)	48 (14.59)	85 (25.84)	168 (51.06)	0.000
Nurses	48 (14–50)	1 (0.26)	4 (1.06)	4 (1.06)	10 (2.65)	17 (4.50)	63 (16.67)	279 (73.81)	
Cleaning workers	50 (41–50)	0 (0)	0 (0)	0 (0)	0 (0)	0 (0)	1 (10.00)	9 (90.00)	
**Length of service**									
<5 years	48 (14–50)	1 (0.50)	3 (1.50)	5 (2.50)	10 (5.00)	20 (10.00)	34 (17.00)	127 (63.50)	0.582
5–10 years	48 (18–50)	1 (0.36)	1 (0.36)	5 (1.81)	11 (3.97)	28 (10.11)	46 (16.61)	185 (66.79)	
>10 years	46.5 (30–50)	0 (0)	0 (0)	3 (1.25)	7 (2.92)	17 (7.08)	69 (28.75)	144 (60.00)	
**Education**									
Bachelor degree or below	48 (14–50)	2 (0.33)	4 (0.65)	10 (1.63)	20 (3.26)	58 (9.45)	122 (19.87)	398 (64.82)	0.109
Master degree or above	46 (28–50)	0 (0)	0 (0)	3 (2.91)	8 (7.77)	7 (6.80)	27 (26.21)	58 (56.31)	
**Hospital Grade**									
Primary	48 (41–50)	0 (0)	0 (0)	0 (0)	0 (0)	0 (0)	3 (21.43)	11 (78.57)	0.001
Secondary	46 (14–50)	2 (0.84)	4 (1.69)	4 (1.69)	14 (5.91)	30 (12.66)	52 (21.94)	131 (55.27)	
Tertiary	48 (26–50)	0 (0)	0 (0)	9 (1.93)	14 (3.00)	35 (7.51)	94 (20.17)	314 (67.38)	

Similar to the above findings about healthcare workers' attitudes, female staff were more active than male staff in carrying out personal protection, patient care, and environmental disinfection practice against SARS-CoV-2 infection (KW = 18.564, *P* < 0.001). Nurses (KW = 6.358, *P* < 0.001, Adj. *P* < 0.001) and cleaning workers (KW = −2.585, *P* = 0.010, Adj. *P* = 0.029) had a higher score than physicians. Medical staff in tertiary hospitals performed better in practice than those in secondary hospitals (KW = −3.591, *P* < 0.001, Adj. *P* = 0.001).

### The Relationships Among Knowledge, Attitudes, and Practice

**Figure 1:**
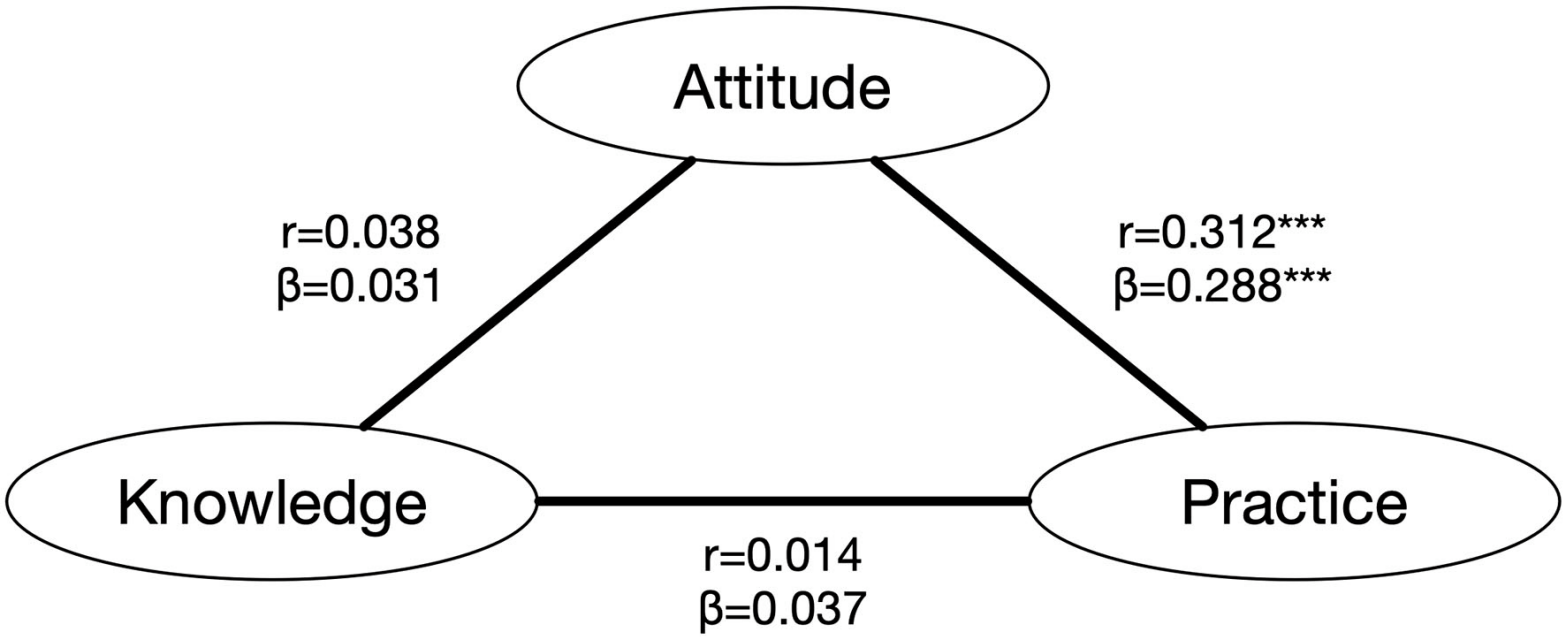
The relationships among knowledge, attitudes, and practice. ***P < 0.001.

The relationships among three dimensions were explored via Spearman's rank correlation analysis. As a result, there was no significant correlation either between knowledge and practice (*r* = 0.014; *P* = 0.710) or between knowledge and attitudes (*r* = 0.038; *P* = 0.314). However, a positive correlation between the level of attitudes and practice was found in the subjects (*r* = 0.312; *P* < 0.001). More positive attitudes in protection were related to better protective behavior in endoscopic daily medical work (Figure 1).

## Discussion

The KAP proposed in the last century has been applied to explaining how personal knowledge and attitudes affected practice in various fields (23–25). In general, knowledge is the basis of behavior formation, and only when knowledge rises to the level of belief can an individual be possible to adopt a positive attitude to change practice. During the COVID-19 pandemic, Chinese health departments have organized various forms of learning activities about SARS-CoV-2, including the virus characteristics, transmission routes, personal protection, quarantine policies, and so on. All the Chinese citizens had access to the knowledge, which was transformed into beliefs. Positive beliefs and attitudes were the motivation for the protective behavior. The medical staff have close contact with patients, and the risks was high, and the KAP theory was more important for medical staff. Therefore, we designed the present questionnaire and enrolled staffs from different institutions to investigate the application of KAP theory by endoscopic healthcare workers in COVID-19 pandemic in China (26–29).

It was found that a high proportion of participants had a good knowledge of COVID-19, which could be possibly attributed to the effective continuing medical education and training going on across the country. Endoscopy-related continuing medical education has an important part to play in preparing for and responding to this situation. Li et al. (30) underscored the importance of continuous medical education and training in this pandemic. Chinese National Health Commission has held online lectures, requiring all medical staff to learn the characteristics and protection requirements of COVID-19.

Moreover, the Endoscopic Society delivered a course of recommended operating procedures in endoscopy units, especially about personal protection and endoscope decontamination, to related healthcare workers, and related questions were required to answer after the course. SARS-CoV-2 is a newly emerged virus, whose virological and disease characteristics are gradually explored and may change at any time. Therefore, continuing education courses for medical staff are also regularly updated in order to enable them to better cope with COVID-19.

Healthcare workers had an extremely positive attitude and carried out favorable practice overall in COVID-19 pandemic. We found that women tended to be more concerned about strengthening the occupational protection, disinfection and management than men, and they did better than men in protective behavior as well. There was a similar phenomenon between nurses and doctors. However, ~87% of men were endoscopy physicians, whereas over 70% of women were nurses in endoscopy units. The results above couldn't distinguish whether the differences in attitudes and behavior were due to gender, occupation, or both of them. We further analyzed the differences between male/female endoscopy physicians and male/female nurses, and noticed that there was a statistical difference between male doctors and female nurses in attitudes. The distinctions in behavior were mainly caused by occupation, not gender. The causes might be as follows. Firstly, nurses spend more time with patients than endoscopy physicians. Endoscopy nurses need to not only assess patients, answer patients' questions and address their concerns before the procedure but also assist doctors throughout the procedure, help patients recover, and complete all necessary documentation including patient notes and discharge documents after the procedure. Secondly, nurses may be more aware of the disinfection because they are responsible for preparing the instruments, equipment and supplies for the procedure as well as cleaning and sterilizing equipment before and after use.

Additionally, medical staff in tertiary hospitals had better protective behavior than those in secondary hospitals. Tertiary hospitals are comprehensive or general hospitals at the city, provincial or national level with a bed capacity exceeding 500. One possible explanation of the phenomenon above is as follows. During the outbreak of COVID-19, it was recommended to defer the elective endoscopies and only perform the urgent endoscopies by strategically assigned staff to minimize concomitant exposure. Endoscopic examinations on patients who were suspected or confirmed with COVID-19 should be performed in a negative pressure room with strict isolation precautions when available (31). Therefore, it was more in line with the protection requirements to complete the urgent endoscopies in a tertiary hospital setting, where the medical staff was more experienced in protective measures and environmental treatment.

The present study investigated the relationships among knowledge, attitudes, and practice of healthcare workers during the prevention and control of new coronavirus infection. The attitudes of endoscopic healthcare workers were positively related to their actual behaviors. In addition, according to theories of mediation effects and KAP, people acquire protection-related knowledge through learning, when their beliefs and attitudes gradually form, which contribute to the emergence of corresponding behavior (32, 33). In this study, we attempted to explore this pattern through mediation effect analysis, but failed to reach a statistical result.

The present study also has some limitations. We only received 10 questionnaires from the cleaning workers, which might be too small to present the real world accurately, thus affecting the comparison among different occupational identities. A larger sample of research is required to be conducted in the future. In addition, our study has geographical bias, to some extent. Most of the questionnaires collected came from non-epidemic areas, while there were fewer questionnaires from areas with severe epidemics. There were particularities in the questionnaire during the epidemic. In the early stage of the epidemic, the country issued corresponding policies that required all organizations to learn the knowledge of the COVID-19, which led to the skewed results of the questionnaire and a narrow gap of knowledge among different occupational identities, thereby concealing some statistical differences.

## Conclusions

In conclusion, most Chinese healthcare workers in endoscopy units are well-trained for protection against COVID-19 infection. Given the adequate protective knowledge, more positive attitudes lead to more effective practice. Female staff has a more positive attitude than male staff, and nurses perform better in both attitudes and practice than endoscopic physicians. Medical staff in tertiary hospitals is more experienced in practice than those in secondary hospitals.

The outbreak of COVID-19 has exposed human vulnerability to unknown diseases, and new viruses have caught us off guard. Future campaigns on medical education should emphasize medical staff's knowledge about the virus and the corresponding protective measures they should take to respond to such sudden public health incidents, especially the protective practice for medical operations, such as endoscopy and endotracheal intubation, which have a high risk of exposing the staff to respiratory infectious diseases.

## Data Availability Statement

The original contributions presented in the study are included in the article/Supplementary Material, further inquiries can be directed to the corresponding author/s.

## Ethics Statement

The studies involving human participants were reviewed and approved by Peking University First Hospital Biomedical Research Ethics Committee (No. 2020-124). Written informed consent for participation was not required for this study in accordance with the national legislation and the institutional requirements.

## Author Contributions

LR had the idea for and took responsibility for the integrity of the data and the accuracy of the data analysis. XG, YT, and YM designed the questionnaire. XG and BN collected the questionnaire and provided the analysis. YT, YM, BN, and XG contributed to the statistical analysis. YT, YM, and BN drafted the manuscript. LR revised the manuscript. FW participated in the literature search and discussion. All authors read and approved the final manuscript.

## Conflict of Interest

The authors declare that the research was conducted in the absence of any commercial or financial relationships that could be construed as a potential conflict of interest.
