# Supplementary material for: The Status of Occupational Protection During COVID-19 Pandemic: Knowledge, Attitudes, and Practice of Healthcare Workers in Endoscopy Units, China
Source: Front Public Health. 2021 Mar 22;9:632608. doi: 10.3389/fpubh.2021.632608 (PMC8019814; doi:10.3389/fpubh.2021.632608)
Supplement: Supplementary file 1 [file Data_Sheet_1.docx]

**Appendix 1 Questionnaire on the status of occupational protection against COVID-19 in endoscopy units**

| **Knowledge Part (Yes; No; Not Clear)** | |
| --- | --- |
| Q1 | The incubation period of COVID-19 is 1 to 14 days, mostly 3-7 days. |
| Q2 | Close contact with COVID-19 patients requires medical observation for 14 days. |
| Q3 | Medical surgical masks generally should be replaced within 4 hours, and should be replaced at any time when contaminated or wet. |
| Q4 | Wearing gloves can replace hand washing. |
| Q5 | When there is no visible contamination after the daily work during the epidemic period, the surface of objects and equipment should be disinfected with 500 mg/L chlorine-containing disinfectant. |
| Q6 | The domestic garbage generated during the examination of patients with pneumonia or suspected pneumonia shall be managed and disposed of as domestic garbage. |
| Q7 | During the epidemic, triage nurses, endoscopy physicians, and cleaning workers need to take different protective measures. |
| Q8 | Patients with pneumonia or suspected pneumonia during the outbreak should be directed to a relatively isolated place for inspection. |
| Q9 | For all patients who have received endoscopy services during the pandemic, the follow-up of their health status within 2 weeks after surgery should be carried out. If there is a patient confirmed with or highly suspected with COVID-19, all personnel contacted during the endoscopic procedure should be traced and isolated. |
| Q10 | The endoscope cleaning fluid needs to be replaced and the cleaning tank needs to be disinfected every time after use. |
| Q11 | There is no need to wear goggles and protective screens at the same time. |
| Q12 | All protective wears except masks should be taken off before leaving the working area. |
|  |  |
| **Attitude Part (strongly agree; agree; neutral; disagree; strongly disagree)** | |
| Q1 | A mastery of necessary knowledge about occupational prevention and control of COVID-19may contribute to reducing the cross infection. |
| Q2 | It is important for the triage nurses to investigate the epidemiology history and body temperature of the patients. |
| Q3 | Endoscopy units need to enhance personal protection and infection control measures against SARS-CoV-2. |
| Q4 | During endoscope operation, to promptly replace the damaged protective equipment is an important measure for occupational prevention and control. |
| Q5 | Being concerned about the use and performance of various disinfectants is important to reduce the risk of infection. |
| Q6 | Timely isolation is important to reduce the risk of infection. |
| Q7 | After endoscopy, the clinic needs to be terminally disinfected. |
| Q8 | Different protective measures for different areas in the endoscopy units are important to reduce the risk of infection. |
| Q9 | During the pandemic, it is necessary for the endoscopy units to limit daily endoscopy services. |
| Q10 | Each endoscope must be disinfected according to the guideline of confirmed cases. |
| Q11 | It is important to avoid physical contact with the patients who have aggressive behavior during pandemic. |
| Q12 | If the patient's body fluid or blood splashes on the protective clothing, replace them immediately. |
| Q13 | Personal protection training in endoscopy units is important. |
|  |  |
| **Practice Part (Always; Often; Sometimes; Occasionally; Hardly ever)** | |
| Q1 | All endoscopy procedures are performed strictly following the guideline during the pandemic. |
| Q2 | Limited daily endoscopic services are provided during the pandemic. |
| Q3 | Occupational protection instructions for healthcare workers are made in endoscopy units. |
| Q4 | Patients undergoing endoscopy during the pandemic are required to receive chest CT or throat swab test for COVID-19 within 3 days. |
| Q5 | All the patients and escorts are required to measure body temperature and be enquired about epidemiological history and medical history before endoscopy. |
| Q6 | Wear surgical masks or N95 masks, gloves, goggles, protective masks, protective clothing, isolation clothing, protective shoe covers, working caps during endoscopic operations. |
| Q7 | Set a fixed area to store tissue specimens obtained during endoscopy procedures. |
| Q8 | The endoscopy units are cleaned and disinfected according to different functional areas. |
| Q9 | Be vigilant when communicating with and performing operations on patients suspected of excessive behavior. |
| Q10 | After the operation, take off protective equipment in the buffer zone and wash your hands or disinfect your hands immediately. |
